# For the Public Good

**Published:** 1907-05

**Authors:** 


### FOR THE PUBLIC GOOD.

An independent and ably edited newspaper which commands a great circulation is probably the most potent influence for good in the United States to-day. The power for the better things in public affairs and policies, for instance, which is wielded by such a newspaper as the Chicago Record-Herald can scarcely be exaggerated, and much of that strength comes in the case of this leading Chicago daily from the fact that it is absolutely independent, fearless and fair. It is not the mouthpiece of any interest except that of the public. The Record-Herald champions the cause of the good, the clean, the beneficial in every matter of city, state or national moment. It is the knowledge on the part of its readers that it cares not whom it hits or what enemies it makes, so long as it is battling for the welfare of the community, which gives to the Record-Herald much of the influence it enjoys. It gives in its news columns the most complete and impartial reports of political events, another evidence of its splendid news service.

